# GBMPhos: A Gating Mechanism and Bi-GRU-Based Method for Identifying Phosphorylation Sites of SARS-CoV-2 Infection

**DOI:** 10.3390/biology13100798

**Published:** 2024-10-06

**Authors:** Guohua Huang, Runjuan Xiao, Weihong Chen, Qi Dai

**Affiliations:** 1 College of Information Science and Engineering, Shaoyang University, Shaoyang 422000, China; guohuahhn@163.com (G.H.); xrj0405@163.com (R.X.); 2 School of Information Technology and Administration, Hunan University of Finance and Economics, Changsha 410205, China; 3 College of Life Science and Medicine, Zhejiang Sci-Tech University, Hangzhou 310018, China; daiqi@zstu.edu.cn

**Keywords:** Bi-GRU, deep learning, gating mechanism, phosphorylation, SARS-CoV-2

## Abstract

**Simple Summary:**

Phosphorylation is a crucial process that regulates various cellular activities. Detecting phosphorylation sites, especially in cells infected by the SARS-CoV-2 virus, is challenging due to technical limitations. To address this, we developed GBMPhos, an advanced tool combining deep learning techniques, to accurately identify these sites. GBMPhos outperformed traditional methods and current state-of-the-art approaches in identifying phosphorylation sites. We have developed a free web server, which helps researchers gain a better understanding of protein modifications during a SARS-CoV-2 infection, potentially aiding in the development of therapeutic strategies and contributing to the fight against COVID-19.

**Abstract:**

Phosphorylation, a reversible and widespread post-translational modification of proteins, is essential for numerous cellular processes. However, due to technical limitations, large-scale detection of phosphorylation sites, especially those infected by SARS-CoV-2, remains a challenging task. To address this gap, we propose a method called GBMPhos, a novel method that combines convolutional neural networks (CNNs) for extracting local features, gating mechanisms to selectively focus on relevant information, and a bi-directional gated recurrent unit (Bi-GRU) to capture long-range dependencies within protein sequences. GBMPhos leverages a comprehensive set of features, including sequence encoding, physicochemical properties, and structural information, to provide an in-depth analysis of phosphorylation sites. We conducted an extensive comparison of GBMPhos with traditional machine learning algorithms and state-of-the-art methods. Experimental results demonstrate the superiority of GBMPhos over existing methods. The visualization analysis further highlights its effectiveness and efficiency. Additionally, we have established a free web server platform to help researchers explore phosphorylation in SARS-CoV-2 infections. The source code of GBMPhos is publicly available on GitHub.

## 1. Introduction

Protein phosphorylation, a post-translational modification (PTM), involves the transfer of a covalently bound phosphate group to an amino acid by the protein kinase. Phosphorylation most frequently occurs at the serine, threonine, tyrosine, and histidine, and it occurs occasionally at the arginine, lysine, aspartic acid, glutamic acid, and cysteine [1,2,3]. Phosphorylation is a reversible process. The reverse reaction, called dephosphorylation, is catalyzed by protein phosphatases. Phosphorylation is the most prevalent post-translational modification, with approximately 13,000 human proteins being phosphorylated and about 41% of translated proteins in Arabidopsis undergoing phosphorylation [4]. This modification can alter protein structural conformation by introducing a charged and hydrophilic group to the side chain of amino acids, acting as a switch to activate or deactivate proteins [5]. For example, the phosphorylation of certain crucial components in plants can switch on or off specific signaling pathways related to growth or defense. Therefore, phosphorylation is essential for the regulation of cell growth, differentiation, apoptosis, and cell signaling [6]. Aberrant phosphorylation is commonly associated with various diseases, such as cancer, diabetes, and developmental defects [7].

The significant advances in proteomics technology, including mass spectrometry and single-molecule techniques, have greatly facilitated the identification of PTM sites [8]. To date, over 400 diverse types of PTMs have been discovered, and more than 262,522 post-translationally modified residues have been annotated [9,10]. Nevertheless, given that proteomics technology is laborious and time-consuming, quickly identifying phosphorylation sites from the huge volume of unknown protein sequences still remains challenging. On the contrary, computational approaches are powerful enough to analyze hundreds of millions of proteins within a short period and are increasingly becoming a complement to proteomics technology in identifying phosphorylation sites. Over the past two decades, at least ten computational methods have been developed for this purpose [11]. For example, Xue et al. developed a group-based prediction system (GPS) for phosphorylation site prediction [12,13]. Wong et al. introduced a profile-hidden Markov model to identify kinase-specific phosphorylation sites. The hidden Markov model is trained using annotated proteins and can then make predictions based on predefined inputs [14]. Wei et al. utilized a powerful representation of sequences from multiple perspectives and developed a random forest-based method for phosphorylation prediction [15]. Traditional machine learning methods relied heavily on representations of protein sequences and the algorithms used. The efficiency and effectiveness of these algorithms in predicting phosphorylation were closely associated with the representations.

In practice, most representations were degenerated. Namely, many diverse protein sequences could correspond to the same or a similar representation. Consequently, traditional machine learning methods often struggled to achieve higher predictive accuracy in most cases. Deep learning (DL) requires no predefined representations and is an end-to-end method with a powerful fitting ability. Hence, it is attracting more and more attention from all trades and professions. Wang et al. introduced MusiteDeep, the inaugural DL approach for predicting kinase-specific phosphorylation sites [16]. MusiteDeep employed convolutional neural networks (CNNs) and attention layers to construct a deep neural network, significantly enhancing the performance of general phosphorylation site prediction. Subsequently, Wang et al. [17] improved MusiteDeep by using the multi-scale CNN and capsule networks [18]. Luo et al. [19] introduced a diverse deep learning framework called DeepPhos for phosphorylation prediction. The DeepPhos used densely connected CNNs, where convolutional layers are connected to improve the representation of sequences. Guo et al. presented two parallel DL modules to extract local and global representations from phosphorylated protein sequences [20]. Each module included two squeeze-and-excitation blocks and one bi-directional long short-term memory (Bi-LSTM) block, with the outputs concatenated to determine phosphorylation [21]. Undoubtedly, DL algorithms have become essential tools for exploring scientific research (AI for science).

In 2019, severe acute respiratory syndrome coronavirus 2 (SARS-CoV-2) quickly spread worldwide. As of 3 March 2024, the world had seen more than 774 million confirmed cases and over 7 million fatalities [22]. SARS-CoV-2 has presented a significant threat to human health and safety. Over the past several years, the relationship between SARS-CoV-2 infection and phosphorylation has been extensively investigated. For instance, Bouhaddou et al. conducted phosphoproteomics research on SARS-CoV-2 infection in Vero E6 cells using mass spectrometry [23]. Similarly, Hekman et al. [24] conducted a quantitative phosphoproteomic survey. Lv et al. [25] employed CNNs and Bi-LSTM to construct a DL architecture for predicting the phosphorylation of host cells infected with SARS-CoV-2. Zhang et al. [26] utilized the attention mechanism and the bi-directional gated recurrent unit (Bi-GRU) network to develop an explainable model for predicting phosphorylation sites associated with SARS-CoV-2 infection.

In this paper, we employed CNNs, the gating mechanism, and Bi-GRU to construct a diverse DL architecture called GBMPhos for identifying phosphorylation sites of host cells infected with SARS-CoV-2. The gating mechanism is designed to control the flow of information, preserving informative representation while discarding less relevant data. GBMPhos takes various encodings of protein sequences as input, including one-hot encoding, BLOSUM62, ZScale, Binary_5bit_type 1, and Binary_5bit_type 2. The combination of CNNs, Bi-LSTM, and the gating mechanism is used to extract high-level representations.

## 2. Materials and Methods

### 2.1. Materials

For machine learning-based methods, selecting appropriate benchmark datasets is crucial for assessing performance. We used datasets compiled by Lv et al. [25] as our benchmark dataset. The reasons for this are expressed as follows: Firstly, all the phosphorylation sites in these datasets were experimentally validated, ensuring high quality. Secondly, it is convenient to compare GBMPhos with existing methods. The process of collecting and preprocessing data is briefly described as follows: Lv et al. first collected 14,119 experimentally confirmed phosphorylation sites from SARS-CoV-2-infected human A549 cells. They then used a sequence clustering software called CD-HIT (v4.8.1) [27] to reduce or eliminate sequence homology and redundancy, setting the sequence identity threshold to 0.3. All protein sequences were further segmented into 33-residue peptides with S/T at the center. Peptides containing phosphorylation sites were labeled as positive samples, while others were labeled as negative samples. Because of the significant imbalance between the number of positive and negative samples, training a classifier on such an unbalanced dataset could prefer negative to positive samples at the stage of prediction. To remove unfavorable effects on phosphorylation prediction, Lv et al. randomly chose the same number of negative samples from the pool of non-phosphorylation peptides, resulting in a balanced dataset comprising 5387 positive and 5387 negative samples. The datasets were randomly divided into a training set and a test set, at a ratio of 8:2.

### 2.2. Methods

**Figure 1:**
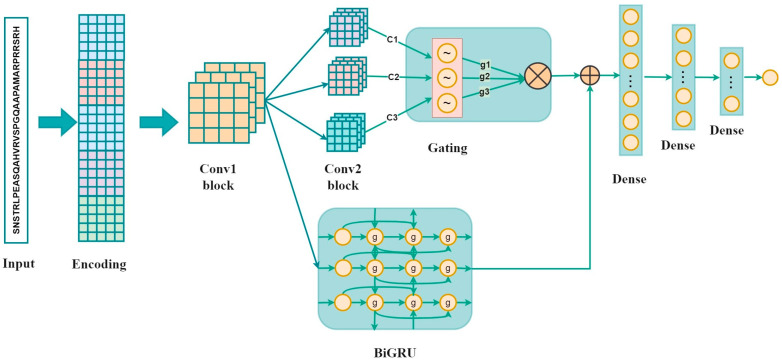
The overall architecture of the GBMPhos. c1, c2, and c3 denote the outputs of the three convolutional layers: g1 = sigmoid (c1), g2 = 1-sigmoid (c2), and g3 = sigmoid (c3); g1, g2, and g3 are the probability values between 0 and 1 by converting the outputs of the three convolutional blocks by the sigmoid function, indicating the importance of the three channels.

As shown in Figure 1, the proposed method is composed of the inputs, sequence encoding, and a convolution layer followed by three parallel convolution layers, a Bi-GRU layer, three fully connected layers, and the output. The inputs, which are 33-residue peptides, were first encoded using one-hot encoding, BLOSUM62, ZScale, Binary_5bit_type 1, and Binary_5bit_type 2. Subsequently, a 1D convolution neural network was used to refine high-level representations. Three parallel CNNs and a Bi-GRU were employed to capture local characteristics and the long-range dependencies, respectively. The outputs of three parallel CNNs were then multiplied by the gating mechanism. The gated representations and the output of the Bi-GRU were fused by element-wise addition operation [28,29]. The final output denoted a neuron which represented the probability of classifying the input as a phosphorylation site.

#### 2.2.1. One-Hot Encoding

One-hot encoding is a straightforward yet effective representation of DNA/protein sequences. For protein sequences comprising 20 amino acids, the one-hot encoding used a 20-dimensional binary vector to represent an amino acid residue, where only a bit is 1, and others are 0. Due to its simplicity and effectiveness, the one-hot encoding was widely applied to the area of bioinformatics, including RNA pseudouridine site identification [30], prediction of nucleosome positioning [31], and high sgRNA on-target activity prediction [32].

#### 2.2.2. BLOSUM62

The BLOSUM62 matrix is a commonly used substitution matrix to measure the ability to substitute between different amino acids. Each element of the matrix represents the score of the *i*-th amino acid substitution for the *j*-th amino acid. The protein primary sequence information was represented using the BLOSUM62 matrix as the basic feature set [33].

#### 2.2.3. ZScale

ZScale is a descriptor of amino acids, which uses five physicochemical values to characterize an amino acid. The ZScale was initially developed by Sandberg et al. in 1998 for the design of biologically active peptides [34]. The ZScale reflected differences and similarities between different amino acids in a protein sequence in a physicochemical respect and thus was used further to analyze the structure and function of the proteins. The ZScale values for 20 amino acids are shown in Table 1.

#### 2.2.4. Binary_5bit_Type 1

A total of 20 types of amino acids are categorized into five groups based on their physicochemical properties. Specifically, the first group is G, A, V, L, M, and I; the second is F, Y, and W; the third is K, R, and H; the fourth is D and E; and the fifth is S, T, C, P, N, and Q. Binary_5bit_type 1 [35,36] used a five-bit binary vector to represent an amino acid. In a five-bit binary vector, each bit reflects the group that the amino acid belongs to. For example, G, A, V, L, M, and I were encoded into (1, 0, 0, 0, 0), while S, T, C, P, N, and Q were encoded into (0, 0, 0, 0, 1).

#### 2.2.5. Binary_5bit_Type 2

For a five-bit binary vector, there are 32 possible combinations. The number of vectors with elements of all the zero or all the one is 2, and the number of vectors whose elements are of only a zero or only a one is 10. Apart from two types of vectors, there are twenty vectors. The Binary_5bit_type 2 used these 20 vectors to represent 20 amino acids [37]. Namely, the Binary_5bit_type 2 encoded A into (0, 0, 0, 1, 1), C into (0, 0, 1, 0, 1), D into (0, 0, 1, 1, 0), E (0, 0, 1, 1, 1), F (0, 1, 0, 0, 1), G (0, 1, 0, 1, 0), H (0, 1, 0, 1, 1), I (0, 1, 1, 0, 0), K (0, 1, 1, 0, 1), L (0, 1, 1, 1, 0), M (1, 0, 0, 0, 1), N (1, 0, 0, 1, 0), P (1, 0, 0, 1, 1), Q (1, 0, 1, 0, 0), R (1, 0, 1, 0, 1), S (1, 0, 1, 1, 0), T (1, 1, 0, 0, 0), V (1, 1, 0, 0, 1), W (1, 1, 0, 1, 0), and Y into (1, 1, 1, 0, 0).

#### 2.2.6. CNN

The CNN is famous as a feed-forward neural network for its powerful ability to especially characterize images. Unlike the multilayer perceptron, the CNN shares weight in a layer via a filter (called convolution kernel). The CNN typically includes a convolution layer and a pooling layer. The former performs a dot product of the filter with a receptive field (element-by-element multiplication sum) and subsequent activation. The dot product is changed with receptive fields when the filter slides along the input, but the filter is identical in the same layer. The pooling layer is a simple subsampled technique. The pooling layer includes the global and local pooling. The global pooling operates on all the neurons, while the local pooling is within a certain size such as the 3 × 3 region. The pooling layer is also divided into average pooling and max pooling. Due to its remarkable success in image recognition [38,39], the CNN is increasingly attracting attention from multiple discipline communities, including bioinformatics. For example, Tang et al. [40] employed the CNN to predict DNA 6 mA sites, Tahir et al. [41] utilized the CNN to detect RNA pseudouridine sites, and Dou et al. [42] used the CNN to classify human nonhistone crotonylation sites. Here, we used one-dimensional CNN.

#### 2.2.7. Bi-GRU

A GRU was introduced in an improved version of an RNN [43]. The GRU uses the update gate and the reset gate to modulate the information flow. The update gate decides how much information in the past is passed to the next step. The update gate is computed by

*z_t_* = *σ*(*W^Z^x_t_* + *U^Z^h*_*t*−1_), (1)

where *h_t_*_−1_ is the hidden state of the (*t* − 1)-th time step. *h_t_*_−1_ represents the information about the previous *t*−1 time steps, *x_t_* is the input of the *t*-th step, and *W^z^* and *U^z^* are the learnable parameters. *σ* is the activation function which is generally set to the sigmoid. The reset gate determines how much information in the past is forgotten, which is computed by

*r_t_* = *σ*(*W^r^x_t_* + *U^r^h*_*t*−1_), (2)

where *W^r^* and *U^r^* correspond to the learnable parameters. The new memory is used to save the previous information, which is computed by

*h*^′^*_t_*= tanh(*Wx_t_* + *r_t_* ⊙ *Uh_t_*_−1_), (3)

where ⊙ denotes the Hadamard (element-wise) product, and W as well as U correspond to the learnable parameters. The hidden state is updated by

*h_t_* = tanh(*z_t_* ⊙ *h_t_*_−1_ + (1 − *z_t_*) ⊙ *h*^′^*_t_*). (4)

The structure of the GRU is similar to that of LSTM: both used a gating mechanism. The main difference from the LSTM is that the GRU lacks the output gate, which results in fewer parameters than the former. The GRU has the potential to solve the issue of vanishing or exploding gradients in the DL field. Here, we used a bi-directional GRU.

#### 2.2.8. Fully Connected Layer

We used three fully connected layers. The first fully connected layer tended to reduce dimensions, which converted high-dimension vectors into one-dimension vectors. The second fully connected layer corresponds to the hidden layer in the multilayer perceptron. The third fully connected layer has one neuron, which represents probabilities of predicting the samples as phosphorylation.

### 2.3. Performance Evaluation

We used a hold-out test to examine the performance and employed the independent test to examine generalization. In the hold-out test, we randomly separated 20% from the training dataset as validation. In the independent test, we used the testing set to examine the trained model. To assess the effectiveness of the proposed method, we utilized standard evaluation metrics such as Accuracy (ACC), Specificity (SP), Sensitivity (SN), and Matthews Correlation Coefficient (MCC) [44,45]. They were defined as follows:

$$SN=\frac{TP}{TP+FN}$$

$$SP=\frac{TN}{FP+TN}$$

$$Precision=\frac{TP}{TP+FP}\;Recall=\frac{TP}{TP+FN}\;ACC=\frac{TP+TN}{TP+FN+FP+TN}$$

$$MCC=\frac{TP\times TN-FP\times FN}{\sqrt{\left(TP+FN\right)\left(TP+FP\right)\left(TN+FN\right)\left(TN+FP\right)}}$$

where *TP*, *FP*, *TN*, and *FN* denote the counts of true positive, false positive, true negative, and false negative, respectively. Additionally, the receiver operating characteristic (ROC) curve, the area under the ROC curve (AUC), and the area under the precision-recall curve (AUPRC) were employed to assess the overall performance, with the area under the receiver operating characteristic curve value closer to 1 indicating superior performance.

## 3. Results

### 3.1. Optimizing of Different Window Sizes

To optimize the window size of sample sequences, we performed experiments with window sizes ranging from 17 to 33. The results are shown in Table 2. The GBMPhos with a window size of 33 achieved an ACC of 0.8528, which was higher than those of other window sizes. Similarly, the window size of 33 also reached the best AUC. Therefore, the window size of 33 demonstrated superior performance over other window sizes. 

### 3.2. Feature Selection

**Figure 2:**
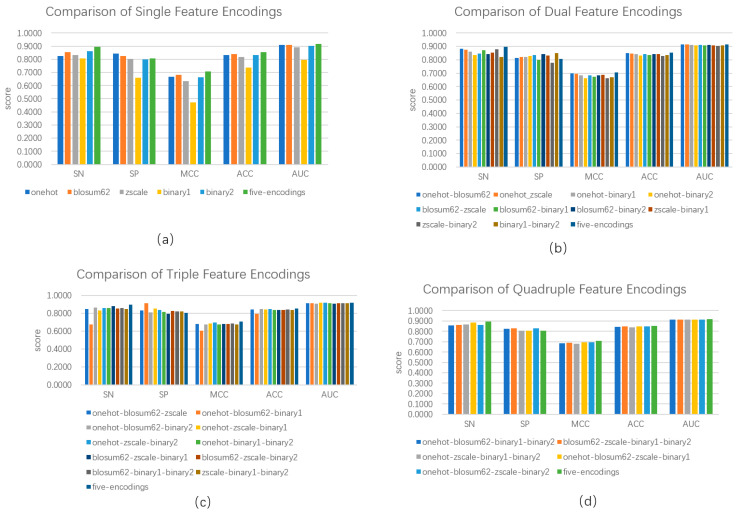
Comparison of model performance based on single feature encoding and combined feature encoding. (**a**) indicates model performance based on a single feature and five features; (**b**) indicates model performance based on two combined features and five features; (**c**) indicates model performance based on three freely combined features and five features; and (**d**) indicates four freely combined features and five features.

We calculated five categories of features, i.e., one-hot encoding, BLOSUM62, ZScale, Binary_5bit_type 1, and Binary_5bit_type 2. We investigated the performance of a single category of features and its combination in distinguishing between phosphorylation and non-phosphorylation using the hold-out test. As shown in Figure 2a, the combination of five categories of features outperformed any single category of features. The combination of all the five categories of feature reached an SN of 0.8953, an SP of 0.8072, an ACC of 0.8528, an MCC of 0.7066, and the AUC of 0.9163, increasing SN by 0.0326, ACC by 0.0115, MCC by 0.0243, and the AUC by 0.0068 over the second best, respectively. Although the one-hot encoding obtained an advantage of SP over the combination, the latter outperformed the former in terms of the other four metrics. Figure 2b demonstrates the performance of a combination of any two categories of features. Combining two categories outperformed five-category combinations in terms of Specificity. Except for this, the five-category combination obtained the best four evaluation metrics. For example, the five-category combination reached an ACC of 0.8528, exceeding 0.003 over the second best. Figure 2c,d show the performances of a combination of any three and any four categories of features, respectively. The performances were very close, and the maximum range did not exceed 0.05. Although the five-category combination was inferior to the combination of three and four in terms of Specificity, its entire performance was still better. Thus, we used five categories of features as representations of protein sequences.

### 3.3. Comparison of Different Structures

The GBMPhos used the Bi-GRU, two CNNs, and a gating mechanism. We examined them for their contributions to discriminating phosphorylation sites using the hold-out test. The tests were conducted under the constraint where only one element was removed or changed, and others were unchanged. Table 3 lists the predictive performances of these tests. Upon removing the Bi-GRU, the GBMPhos performed extremely poorly, achieving an ACC of 0.4832. This was almost equal to the random guess. The replacement of the Bi-GRU with the Bi-LSTM decreased SN by 0.0371, ACC by 0.0181, MCC by 0.0366, and AUC by 0.006 over the GBMPhos. The removal of the first CNN reduced SN by 0.0395, ACC by 0.0133, MCC by 0.028, and AUC by 0.0034 over the GBMPhos. The exclusion of the second CNN reduced SN by 0.0313, ACC by 0.0151, MCC by 0.0313, and AUC by 0.0087. The GBMPhos without the gating mechanism showed a slight decline. For example, SN declined from 0.8953 to 0.8349, ACC declined from 0.8528 to 0.8383, MCC declined from 0.7066 to 0.6766, and the AUC declined from 0.9163 to 0.9113. The empirical tests above indicated the rationality of the GBMPhos.

### 3.4. Parameter Optimization

In the GBMPhos, many hyper-parameters are not learnable but need manual settings, such as the size of the convolution kernel in the CNNs. We optimized these parameters by conducting a hold-out test. We compared four types of kernel sizes in the first convolution layer. As illustrated in Table 4, the convolution with the kernel size of one reached the best Accuracy, the best Matthews Correlation Coefficient, and the best area under the receiver operating characteristic curve. Therefore, we fixed the kernel size of the first convolution to one. There were three convolutions in the second convolution layer. We compared eight combinations of three kernel sizes. The Conv2 (7, 9, 11) obtained the best Specificity but was inferior to the Conv2 (3, 3, 3) in terms of Sensitivity, Accuracy, Matthews Correlation Coefficient, and area under the receiver operating characteristic curve. The Conv2 (5, 7, 9) performed closely with the Conv2 (3, 3, 3). The Conv2 (5, 7, 9) reached a better area under the receiver operating characteristic curve and Specificity than the Conv2 (3, 3, 3). On the other hand, the Conv2 (3, 3, 3) had better Sensitivity, Accuracy, and Matthews Correlation Coefficient than the Conv2 (5, 7, 9). Therefore, we finally decided to use a homoscale convolution with a convolution kernel size of three.

### 3.5. Comparison with Existing Algorithms

We compared the GBMPhos with traditional machine learning algorithms, such as Decision Tree (DT), Logistic Regression (LR), Random Forest (RF), Support Vector Machine (SVM), Extreme Gradient Boosting (XGBoost), Gradient Boosted Decision Trees (GBDT), and Light Gradient Boosting Machine (LGBM) [33]. These algorithms were widely applied to the areas of molecular biology, drug design, and speech recognition. For a fair comparison, we used one-hot encoding, BLOSUM62, ZScale, Binary_5bit_type 1, and Binary_5bit_type 2 of protein sequences as input to these learning algorithms. As listed in Table 5, the GBMPhos outperformed all the traditional learning algorithms. The GBMPhos obtained an SN of 0.8513, an SP of 0.8500, an ACC of 0.8506, an MCC of 0.7010, and an AUC of 0.9209, exceeding the second best by 0.0367 SN, by 0.0241 SP, by 0.0302 ACC, by 0.0604 MCC, and by 0.0174 AUC. 

### 3.6. Comparison with Existing Methods

We compared the GBMPhos with three state-of-the-art methods using the independent test, namely IPs-GRUAtt [26], DeepIPs [25], and Adapt-Kcr [9]. As listed in Table 6, the GBMPhos reached the best Sensitivity, the best Accuracy, the best Matthews Correlation Coefficient, and the best area under the receiver operating characteristic curve. Although the Adapt-Kcr and the IPs-GRUAtt are 0.0072 and 0.0045 more for Specificity than the GBMPhos, respectively, the latter significantly outperformed the two formers in terms of Sensitivity, Matthews Correlation Coefficient, Accuracy, and area under the receiver operating characteristic curve. For example, the GBMPhos had 0.0447 ACC, 0.0174 ACC, and 0.0044 ACC more than the DeepIPs, the Adapt-Kcr, and the IPs-GRUAtt, respectively. Therefore, GBMPhos outperformed three state-of-the-art methods in identifying phosphorylated S/T sites. Additionally, we compared the GBMPhos with the state-of-the-art methods on the tyrosine phosphorylation dataset [25]. The performances are shown in Table 7.

To further show the ability to identify phosphorylated S/T sites, we retrieved a true protein sequence associated with SARS-CoV-2 infection from the UniProt database for testing. The true protein Q96P20 was obtained by performing searches with the following keywords: phosphorylation, post-translational modifications, and modified residue. As shown in Table 8, the Q96P20 contained 12 true phosphorylated S/T sites. The GBMPhos correctly identified four phosphorylated S/T sites (Sensitivity is 1/3) but was wrong in identifying three false phosphorylation sites (Precision is 4/7). As a comparison, we used three other web server-based methods: MusiteDeep2020 (https://www.musite.net/ (accessed on 1 December 2023)), DeepIPs (http://lin-group.cn/server/DeepIPs/Pre-new.php (accessed on 1 December 2023)), and IPs-GRUAtt (http://cbcb.cdutcm.edu.cn/ phosphory/result/ (accessed on 1 December 2023)). The MusiteDeep was right in identifying five phosphorylated S/T sites (Sensitivity is 5/12) and wrong in identifying five phosphorylated S/T sites (Precision is 1/2). The DeepIPs obtained only a Sensitivity of 1/3 and a Precision of 4/9. The IPs-GRUAtt reached only a Sensitivity of 1/6 and a Precision of 2/5. The GBMPhos outperformed these three methods.

### 3.7. Test on Different Positive-to-Negative Sample Ratios

To investigate the impact of positive-to-negative sample ratios on the performance of GBMPhos, we experimented with a ratio of 1:1, as well as the imbalanced dataset including ratios of 1:2, 1:3, 1:4, 1:5, 1:6, 1:7, 1:8, 1:9, and 1:10. We sampled negative samples from the Uniprot database. The results of these experiments are detailed in Table 9. 

As illustrated in Table 9, the GBMPhos achieved an ACC of 0.8506, an SP of 0.8500, an AUC of 0.9209, and an AUPRC of 0.9245 when using a positive-to-negative sample ratio of 1:1. As the proportion of positive-to-negative samples increased, the values of SP, ACC, AUC, and AUPRC generally exhibited a decreasing trend. Although the AUC also showed a decreasing trend with an increase in the proportion of negative samples, it remained above 0.8900 overall. However, when the positive-to-negative sample ratio was 1:10, the AUPRC value decreased to 0.5608, which is lower than the AUC. As the number of negative samples increased, the FP began to exceed the TP. Consequently, the precision, calculated as Precision = TP/(TP + FP), decreased continuously. This was the reason for the decrease of the AUPRC when the ratio of negative samples increased. 

### 3.8. Visualization Analysis

**Figure 3:**
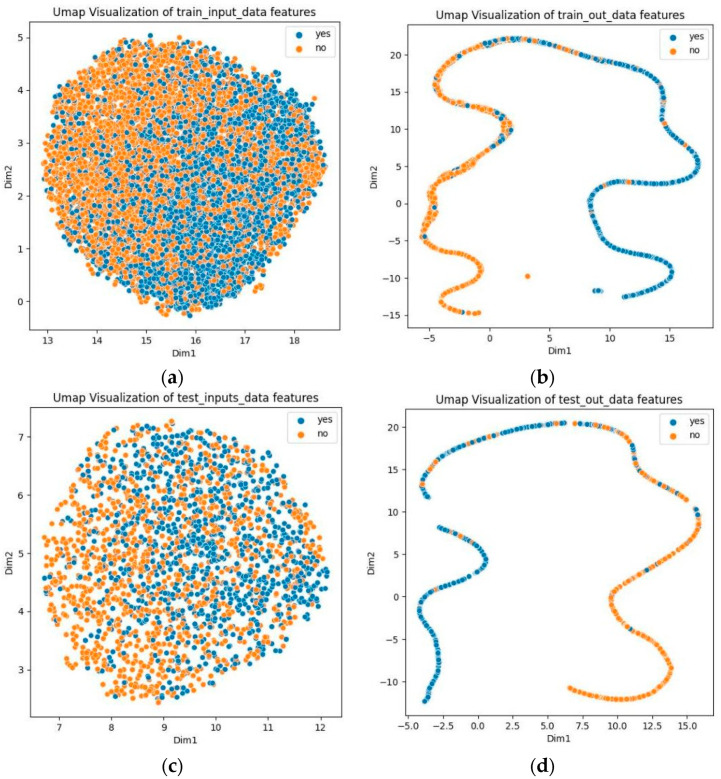
Dataset visualization. “Yes” indicates phosphorylation sites, and “no” indicates no phosphorylation sites; (**a**) indicates visualization of original training data, (**b**) indicates visualization of training data after model output, (**c**) indicates visualization of original test data, and (**d**) indicates visualization of test data after model output.

To demonstrate the capability of predicting phosphorylation sites, we used Uniform Manifold Approximation and Projection (UMAP) to visualize the prediction results for both the training and test sets [46]. As illustrated in Figure 3, the UMAP plots of the original data show difficulty in differentiating between positive and negative samples. However, with GBMPhos, positive and negative samples are separated. These results suggest that GBMPhos is highly effective in identifying phosphorylation sites.

### 3.9. GBMPhos Web Server

**Figure 4:**
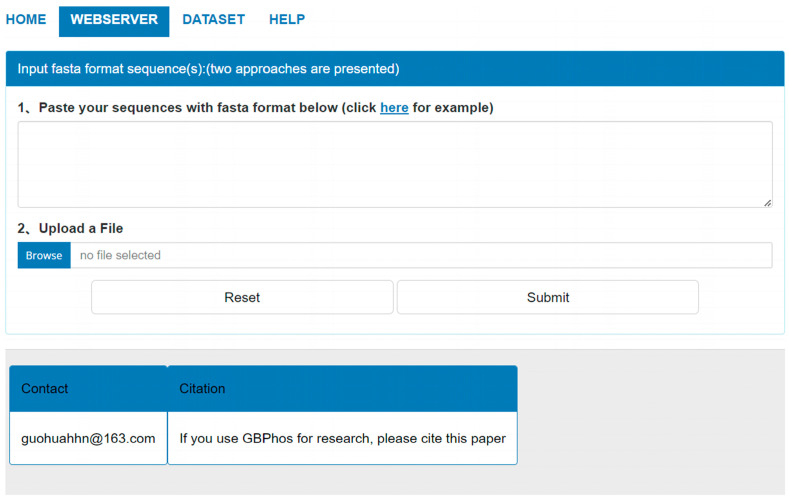
The web server page of GBMPhos.

To facilitate using GBMPhos more conveniently, we developed a user-friendly web server, which is freely accessible at http://www.biolscience.cn/GBPhospred/ (Access on 1 January 2024). As shown in Figure 4, the GBMPhos web server is easy to use. The first step is to submit the sequence to the GBMPhos web server. Sequences in FASTA format can be submitted in two ways: either by pasting the sequence directly into the textbox or by uploading a file. The second step is clicking the submit button to perform a prediction. The prediction results were returned to users from the GBMPhos on the web page. The computational cost was proportional to the number of submitted sequences. In addition, users may re-submit sequences by clicking the Reset button. Users may download all the experimental datasets by clicking the DATASET option.

## 4. Discussion

Rapidly and accurately identifying phosphorylation sites is still a challenging task. We proposed a gating mechanism and a Bi-GRU-based deep learning method to predict S/T phosphorylation sites of SARS-CoV-2 infection. In the independent tests, the GBMPhos achieved better performance than state-of-the-art methods. The GBMPhos used five types of representation of protein sequence as input, i.e., one-hot encoding, BLOSUM62, ZScale, Binary_5bit_type 1, and Binary_5bit_type 2. The DeepIPs used embedding and three pre-trained word embeddings (Word2Vec [47], GloVe [48], and fastText [49,50]) to represent protein sequences. The IPs-GRUAtt only used embedding to learn a representation of proteins. Embedding and pre-trained word embedding could learn the semantics of protein sequences. BLOSUM62, ZScale, Binary_5bit_type 1, and Binary_5bit_type 2 not only represented the composition of amino acids but also characterized physiochemical and evolutionary properties. The GBMPhos could learn the semantics in the one-encoding, the BLOSUM62, the ZScale, the Binary_5bit_type 1, and the Binary_5bit_type 2 by the Bi-GRU. The DeepIPs accumulated repeated homogeneous semantics, the IPs-GRUAtt used only a single type of semantics, and the GBMPhos fused physiochemical and evolutionary properties and semantics. Therefore, from the viewpoint of the information, the GBMPhos is superior to the DeepIPs and the IPs-GRUAtt. The DeepIPs used the CNN and the Bi-LSTM in a series-connection way, which is adverse to accumulating representations learned by the CNN and the LSTM, while the GBMPhos used the CNN and Bi-GRU in a paralleling way, which preserved representations learned by the CNN and the Bi-GRU. In addition, GBMPhos used the gating mechanism to filter out non-essential information, which promoted representations of protein sequences. However, unlike IPs-GRUAtt, GBMPhos failed to explain the sequence patterns involved in phosphorylation.

## 5. Conclusions

We proposed a gating mechanism-based approach for identifying protein phosphorylation sites in SARS-CoV-2 infections, with its advantages summarized as follows: First, semantics were refined from the physiochemical and evolutionary properties rather than from primary protein sequences, enhancing the representation of protein sequences. Secondly, the parallel connection of the CNN and Bi-GRU was beneficial to cumulate both representations. Thirdly, the gating mechanism preserved key information and filtered out irrelevant information by selectively controlling the flow of information, thereby improving the model’s performance. Like most machine learning methods, the proposed method is not easily interpretable. Therefore, future work will explore the use of large language models to enhance interpretability.

## Figures and Tables

**Table 1:** ZScale for 20 amino acids.

Amino Acid	Z1	Z2	Z3	Z4	Z5
A	0.24	−2.23	0.60	−0.14	1.30
C	0.84	−1.67	3.71	0.18	−2.65
D	3.98	0.93	1.93	−2.46	0.75
E	3.11	0.26	−0.11	−3.04	−0.25
F	−4.22	1.94	1.06	0.54	−0.62
G	2.05	4.06	0.36	−0.82	−0.38
H	2.47	1.95	0.26	3.90	0.09
I	−3.89	−1.73	−1.71	−0.84	0.26
K	2.29	0.89	−2.49	1.49	0.31
L	−4.28	−1.30	−1.49	−0.72	0.84
M	−2.85	−0.22	0.47	1.94	−0.98
N	3.05	1.60	1.04	−1.15	1.61
P	−1.66	0.27	1.84	0.70	2.00
Q	1.75	0.50	−1.44	−1.34	0.66
R	3.52	2.50	−3.50	1.99	−0.17
S	2.39	−1.07	1.15	−1.39	0.67
T	0.75	−2.18	−1.12	−1.46	−0.40
V	−2.59	−2.64	−1.54	−0.85	−0.02
W	−4.36	3.94	0.59	3.44	−1.59
Y	−2.54	2.44	0.43	0.04	−1.47

**Table 2:** Comparison of different window sizes.

Window Size	SN	SP	ACC	MCC	AUC
17	0.8253	0.7997	0.8131	0.6253	0.8785
19	0.8195	0.7922	0.8065	0.612	0.8814
21	0.8655	0.7657	0.8179	0.6359	0.8875
23	0.8230	0.8401	0.8311	0.6624	0.8948
25	0.8460	0.8111	0.8293	0.6578	0.9028
27	0.8494	0.8136	0.8323	0.6638	0.9005
29	0.8667	0.7746	0.8227	0.6453	0.8957
31	0.8448	0.8186	0.8323	0.6638	0.9038
33	0.8953	0.8072	0.8528	0.7066	0.9163

**Table 3:** Performance of different model structures.

Method	SN	SP	ACC	MCC	AUC
BiLSTM	0.8582	0.8109	0.8347	0.6700	0.9103
Without Bi-GRU	0	1	0.4832	nan	0.5000
Without Conv1	0.8558	0.8221	0.8395	0.6786	0.9129
Without Conv2	0.8640	0.8097	0.8377	0.6753	0.9076
Without gating	0.8349	0.8420	0.8383	0.6766	0.9113
Bi-GRU	0.8953	0.8072	0.8528	0.7066	0.9163

Note: “nan” refers to an arithmetic error.

**Table 4:** Performance of different model parameters.

Method	SN	SP	ACC	MCC	AUC
Conv1 (1)	0.8953	0.8072	0.8528	0.7066	0.9163
Conv1 (3)	0.8535	0.8231	0.8383	0.6762	0.9071
Conv1 (5)	0.8756	0.7823	0.8305	0.6620	0.9033
Conv1 (7)	0.8558	0.8010	0.8293	0.6584	0.9030
Conv2 (3, 5, 7)	0.8674	0.8122	0.8407	0.6813	0.9108
Conv2 (1, 3, 5)	0.8663	0.8134	0.8407	0.6813	0.9095
Conv2 (5, 7, 9)	0.8872	0.8122	0.8510	0.7024	0.9167
Conv2 (7, 9, 11)	0.8140	0.8619	0.8371	0.6757	0.9103
Conv2 (1, 1, 1)	0.8415	0.8267	0.8341	0.6683	0.9051
Conv2 (5, 5, 5)	0.8391	0.8255	0.8323	0.6646	0.9023
Conv2 (7, 7, 7)	0.8117	0.8485	0.8299	0.6605	0.8941
Conv2 (3, 3, 3)	0.8953	0.8072	0.8528	0.7066	0.9163

**Table 5:** Comparison with machine learning methods on the S/T data.

Method	SN	SP	ACC	MCC	AUC
LR	0.8004	0.7770	0.7884	0.5772	0.8687
DT	0.7120	0.6736	0.6923	0.3857	0.6928
SVM	0.8089	0.7987	0.8037	0.6075	0.8827
RF	0.7243	0.8531	0.7903	0.5832	0.8715
GBDT	0.7861	0.8286	0.8079	0.6156	0.8916
XGB	0.8108	0.8178	0.8144	0.6286	0.8928
LGBM	0.8146	0.8259	0.8204	0.6406	0.9035
GBMPhos	0.8513	0.8500	0.8506	0.7010	0.9209

**Table 6:** Comparison with state-of-the-art methods on the S/T testing dataset.

Method	SN	SP	ACC	MCC	AUC
DeepIPs	0.8007	0.8109	0.8059	0.6117	0.8926
Adapt-Kcr	0.8090	0.8572	0.8332	0.6670	0.9120
IPs-GRUAtt	0.8378	0.8545	0.8462	0.6924	0.9187
GBMPhos	0.8513	0.8500	0.8506	0.7010	0.9209

**Table 7:** Comparison with state-of-the-art methods on the tyrosine phosphorylation dataset.

Method	SN	SP	ACC	MCC	AUC
DeepIPs	0.9048	0.8095	0.8333	0.7175	0.9252
IPs-GRUAtt	0.9524	0.9048	0.9286	0.8581	0.9206
DeepPSP	0.9524	0.5714	0.7619	0.5665	0.8209
GBMPhos	0.9333	0.8800	0.9000	0.7965	0.9000

**Table 8:** Prediction results of different methods on protein Q96P20.

Phosphorylated S/T Sites	Method	Predicted Sites
5, 161, 163, 198, 201, 295, 334, 728, 735, 806, 975, 1035	MusiteDeep	96, 163, 198, 201, 555, 678, 749, 752, 975, 1035
	DeepIPs	102, 163, 198, 201, 334, 387, 740, 742, 1016
	IPs-GRUAtt	102, 198, 201, 387, 680
	GBMPhos	102, 163, 198, 201, 334, 387, 680

**Table 9:** Comparison with different positive-to-negative sample ratios.

Ratio	SP	ACC	AUC	AUPRC
1:1	0.8500	0.8506	0.9209	0.9245
1:2	0.8208	0.8303	0.9025	0.8119
1:3	0.8152	0.8235	0.9018	0.7507
1:4	0.8111	0.8186	0.9007	0.7051
1:5	0.8086	0.8153	0.8988	0.6574
1:6	0.8051	0.8113	0.8974	0.6232
1:7	0.8016	0.8074	0.8964	0.5887
1:8	0.7977	0.8033	0.8956	0.5608
1:9	0.794	0.7994	0.8946	0.5386
1:10	0.7898	0.7950	0.8934	0.5129
Mean and Standard Deviation	0.8049 ± 0.0101	0.8116 ± 0.0115	0.8979 ± 0.0032	0.6674 ± 0.1314

## Data Availability

The data supporting the findings of this study are available at http://www.biolscience.cn/GBPhospred/data/ (Access on 1 January 2024). We encourage researchers to access and utilize these data for further analysis and exploration. All relevant datasets generated or analyzed during this study have been publicly archived and are accessible through the provided link. Additionally, the source code and related resources for this study are available on GitHub at https://github.com/Xiaorunjuan0405/GBMPhos (Access on 1 January 2024).
